# *Tsogolo la Thanzi*: A Longitudinal Study of Young Adults Living in Malawi's HIV Epidemic

**DOI:** 10.1111/sifp.12080

**Published:** 2019-01-28

**Authors:** Sara Yeatman, Abdallah Chilungo, Sydney Lungu, Hazel Namadingo, Jenny Trinitapoli

## Abstract

*Tsogolo la Thanzi (TLT)* was designed to study how young adults navigate sexual relationships and childbearing during a generalized HIV epidemic. TLT began in 2009 with a population‐representative sample of 1,505 women and 574 men between the ages of 15 and 25 living in Balaka, southern Malawi, where regional adult HIV prevalence then stood at 15 percent. The first phase (2009–11) included a series of eight interviews, spaced four months apart. During this time, women's romantic and sexual partners enrolled in the study on an ongoing basis. A refresher sample of 315 women was added in 2012. Seventy‐eight percent of respondents were re‐interviewed in the second phase of TLT (2015), which consisted of follow‐up interviews approximately 3.5 years after the previous interview (ages 21–31). At each wave, detailed information about fertility intentions and behaviors, relationships, sexual behavior, health, and a range of sociodemographic and economic traits was gathered by means of face‐to‐face surveys. Biomarkers for HIV and pregnancy were also collected. Distinguishing features include: a population‐representative sample, closely spaced data collection, dyadic data on couples over time, and an experimental approach to HIV testing and counseling. Data are available through restricted data‐user agreements managed by Data Sharing for Demographic Research (DSDR) at the University of Michigan.

T*sogolo la Thanzi (TLT)* is a six‐year (2009–15) population‐based, longitudinal study of more than 3,000 young adults in high‐HIV‐prevalent, high‐fertility southern Malawi. Tsogolo la Thanzi means “Healthy Futures” in the Chichewa language. The study is comprised of two phases: TLT‐1 (2009–11) and TLT‐2 (2015).

TLT‐1 was designed to understand how young adults in Malawi navigate childbearing and related life‐course events while avoiding HIV or contending with its consequences. The study focused on young men and women aged 15–25 in 2009 who comprise a birth cohort that grew up during the HIV epidemic and suffered particularly high rates of orphanhood (Monasch and Boerma 2004; Hosegood et al. 2007). Structured to capture changes in HIV risk, childbearing goals, and overall well‐being during a dynamic period of the life course, TLT‐1 features closely spaced (tri‐annual) interviews over three years (Figure 1).

**Figure 1:**
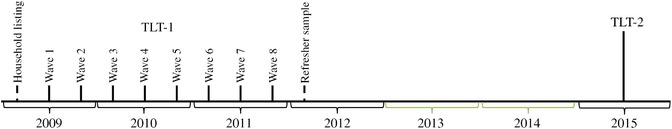
Timeline of TLT‐1 and TLT‐2 (2009–15)

The study team fielded a follow‐up survey (TLT‐2) in 2015 to examine how the relationships between HIV and fertility identified in TLT‐1 might be changing in light of the rapidly changing HIV‐treatment context. To illustrate the magnitude of these changes, rates of HIV testing rose nationally from 7 percent and 15 percent of women and men, respectively, reporting ever having been tested for HIV in 2004 to 83 percent of women and 70 percent of men in 2015 (NSO and ORC Macro 2005; NSO [Malawi] and ICF International 2016). Changes with respect to treatment were equally dramatic over the study period. In 2011, Malawi became the first country in sub‐Saharan Africa to implement Option B+, a test‐and‐treat policy under which all HIV‐positive pregnant and breastfeeding women, regardless of CD4 count (which checks how well the immune system is functioning), were eligible to start lifelong antiretroviral treatment (ART) (Schouten et al. 2011). The number of Malawians on ART more than quadrupled from fewer than 200,000 in 2009 to almost 875,000 in 2015 (Jahn et al. 2016). Based on early indications of success in Malawi, WHO recommended Option B+ as global policy in 2012, and since then 20 other African countries have adopted the policy (WHO 2012; UNAIDS 2016).

## ETHICAL APPROVAL

TLT was set up as a collaboration between researchers at The Pennsylvania State University, the University of Colorado Denver, the University of Chicago, Kamuzu College of Nursing, and the Invest in Knowledge Initiative in Malawi. Ethical approval was obtained in Malawi from the National Health Sciences Research Committee (NHSRC) and in the United States from the Office for Research Protections at The Pennsylvania State University and the Social and Behavioral Sciences Institutional Review Board at the University of Chicago.

## STUDY SETTING

Malawi is a pronatalist country wherein childbearing is critically important to achieving full adulthood (Yeatman and Trinitapoli 2013). The median age of first birth for women is 19 years and the total fertility rate (TFR) surpassed five children per woman as late as 2010 (NSO [Malawi] and ICF Macro 2011 and 2016; Yeatman and Trinitapoli 2013). Malawi also has a generalized HIV epidemic where, as in the rest of sub‐Saharan Africa, HIV is primarily transmitted through heterosexual sex. This poses a set of critical dilemmas for young adults who want children but are also concerned about HIV infection. The risks of contracting HIV and becoming pregnant are synchronized; many behaviors (e.g., abstinence, coital frequency, and condom use) that affect one also affect the other. Not coincidentally, peak HIV incidence for women overlaps almost exactly with peak fertility, underscoring the causal tangle that characterizes this period of the life course.

TLT is based in and around the town of Balaka in southern Malawi, the region of the country with the highest HIV prevalence—about 15 percent at the time TLT began (NSO [Malawi] and ICF Macro 2011). A growing town and district capital, Balaka is located approximately 75 miles (120 kilometers) from Blantyre, along Malawi's main road and trading route, which bisects the country and connects Balaka to trading centers in Mozambique, Tanzania, and Zambia. Balaka is characterized by a religiously and ethnically diverse population surrounded by communities that rely primarily upon subsistence agriculture.

## SAMPLE

TLT's core sample consists of population‐representative samples of young women and young men between the ages of 15 and 25 in 2009; a separate sample of male partners was enrolled and followed over time (Table 1).

**Table 1:** Sample size across study waves, TLT‐1 (2009–11) and TLT‐2 (2015)

	Core sample		Male partners	
Wave	Women	Men	New^a^	Old^b^
1	1,505	574	433	—
2	1,413	542	184	410
3	1,371	524	107	567
4	1,349	506	60	636
5	1,280	472	68	636
6	1,244	464	64	683
7	1,222	437	33	705
8	1,212	441	15	739
Refresher sample^c^	315	—	—	—
TLT‐2	1,453	407	262	311

a Male partners who were newly enrolled in TLT at that wave.

b Previously enrolled male partners completing a follow‐up survey at that wave.

c Refresher sample women added in 2012 and followed up in 2015 as part of TLT‐2.

— = Not applicable.

### Core Sample

In April 2009, TLT conducted a complete household listing of all individuals whose usual residence was within a 4‐mile (7‐kilometer) radius of Balaka's main market. The catchment area includes a train station, a bus depot, a hospital and two smaller district health centers, a cotton “ginnery,” dozens of religious congregations, and about 100 villages of various sizes. The listing exercise enumerated urban town residents and residents of rural villages well outside of town, and went beyond the listing of regular households and their members to include all individuals living in boarding schools and “rest houses” (motels used as residences, often for sex workers) within the catchment area. The core sample was drawn as a simple random sample of 1,562 women between the ages of 15 and 25 in the catchment area of whom 1,505 completed a baseline interview (96.4 percent response rate). Reasons for nonparticipation were refusal (N=30), not found (N=17), and language or mental health problems (N=10). Out of 614 men sampled in this same age‐range, 574 (93.5 percent) completed a baseline survey. Reasons for nonparticipation among men included: not found (N=21), refusal (N=11), and language or mental health problems (N=8).

TLT was powered to study the relationship between childbearing and HIV among women. The smaller core sample of men facilitates comparisons by gender at this same stage of the life course and provides a benchmark for understanding the selective aspects of the male‐partner sample (described below). The random sample of men also provided some “cover” for the romantic partners enrolled in the study; without them, it would have been too obvious that the men in the study had all gotten there because of their relationships with female respondents.

Table 2 provides a descriptive overview of the core sample, by gender, at baseline. Core‐sample respondents come from a number of ethnic groups, the majority of which are traditionally matrilineal and matrilocal (e.g., Lomwe, Ngoni, and Yao), making the TLT sample more matrilocal than the country as a whole, which is approximately split between traditionally matrilineal and traditionally patrilineal ethnic groups (NSO [Malawi] and ICF Macro 2011). There is also considerable religious diversity in the sample: Catholic, Muslim, Mission Protestant, Pentecostal, and New Mission Protestant groups are all well represented. As expected for this age range, women are more likely than men to be married and have children and less likely to be in school.

**Table 2:** Sociodemographic characteristics and retention of core sample by gender, TLT‐1 and TLT‐2

	Women			Men		
	Baseline characteristics	Sample retention		Baseline characteristics	Sample retention	
	Wave 1 N=1,505	Wave 8 N=1,212	TLT‐2 N=1,200	Wave 1 N=574	Wave 8 N=441	TLT‐2 N=407
	%	% retained	% retained	%	% retained	% retained
Overall	100.0	80.5	79.7	100.0	76.8	70.9
Age						
15–19	50.3	77.9*	79.1	57.7	79.8	72.8
20–25	49.7	83.2	80.4	42.3	72.8	68.3
Ethnic group						
Ngoni	38.1	84.7***	84.5***	39.6	84.1*	75.3*
Yao	27.3	81.8	81.8	30.5	73.7	74.9
Lomwe	16.5	76.7	74.3	13.9	67.5	60.0
Chewa	12.1	76.4	75.8	11.0	73.0	61.9
Other	5.9	67.4	62.9	5.1	72.4	62.1
Born in Balaka						
Yes	61.7	85.5***	85.4***	64.6	83.0***	75.7**
No	38.3	72.6	70.7	35.4	65.5	62.1
Religious affiliation						
Catholic	32.3	82.1	82.5	29.6	78.8	74.7
Muslim	20.3	81.7	81.1	23.0	73.5	70.5
Mission Protestant	14.8	74.9	79.8	13.6	74.4	69.2
Pentecostal	14.3	79.1	73.0	17.4	80.0	70.0
New Mission Protestant	13.4	81.1	77.1	11.3	75.4	63.1
Other	4.9	85.1	82.4	5.1	79.3	75.9
Education						
No secondary school	63.9	85.3***	82.5***	56.6	82.5***	76.0**
Secondary school or higher	36.2	72.1	74.8	43.4	69.5	64.3
Enrolled in school						
Yes	38.8	75.5***	76.7*	61.7	79.7*	72.3
No	61.2	83.7	81.7	38.3	72.3	68.6
Marital status						
Never married	50.2	75.0***	77.5	84.2	76.0	71.0
Married	42.1	85.8	81.6	15.3	81.8	70.5
Formerly married	7.6	87.8	84.4	0.5	66.7	66.7
Ever had sex						
Yes	71.6	82.7**	80.3	71.6	75.4	69.1
No	28.4	75.0	78.3	28.4	80.4	75.5
Number of living children						
0	50.6	76.0***	77.8	86.4	75.8	70.0
1	26.4	82.1	79.9	10.5	81.7	78.3
2+	23.0	88.7	83.8	3.1	88.9	72.2
Pregnant^a^						
Yes	12.2	87.5*	83.2	–	–	–
No	87.8	79.6	79.3	–	–	–

NOTE: TLT‐2 figures do not include refresher sample.

a Pregnancy measured using pregnancy test in combination with self‐report in cases of refusal.

Significant at ^***^p<0.001; ^**^p<0.01; ^*^p<0.05.

### Male Partners

Because both HIV infection and pregnancy are dyadic phenomena, TLT was designed with a focus on couple‐level dynamics. Many of the behaviors of interest occur before marriage, thus TLT widened its lens to include not just spouses of women in the sample (a common approach in couples’ studies) but also women's partners at much earlier stages of a relationship. During their first interview, and at all subsequent interviews, women answered detailed questions about their sexual and romantic relationships and then recruited the male partners they named into the TLT study by providing them with tokens that had unique identifiers. Female respondents shared these tokens with their male partners, and the men subsequently enrolled themselves in the study by coming to the TLT research center (N=1,226). Research staff confirmed the relationship through an initial conversation with the male partner at the reception area in the research center and then again based on his reports about his relationships during the survey itself. On a few occasions, the data from male partners were excluded from the study when they were identified as “imposters”—such as one case in which the respondent had passed the token to her brother, rather than her romantic partner. Male partners had a median age of 26 years at enrollment and ranged in age from 13–62.

### Refresher Sample

Immediately following Wave 8, the study team added a refresher sample of 315 female respondents (out of 527, response rate: 60 percent) to TLT‐1. Drawn from the original 2009 sampling frame to offset attrition, the refresher sample also provides a comparison sample against which analysts can identify potential panel‐conditioning effects within the study.

## STUDY DESIGN

TLT‐1 (2009–11) featured an intensive design wherein the core sample of female and male respondents were interviewed every four months for eight waves (Figure 1). The design was motivated by the observation that many critical life events occur during early adulthood (e.g., sexual onset, marriage, school dropout, pregnancy, HIV infection), which makes it difficult (if not impossible) to untangle the temporal ordering of these events using cross‐sectional data or prospective studies with multiyear intersurvey intervals. TLT's closely spaced intervals facilitate the identification of causal relationships during this eventful period of the life course and allow for new insights into the dynamics by which preferences, perceptions, and behaviors change.

The TLT‐2 follow‐up consisted of a single interview in 2015. TLT‐2 included core‐sample women, core‐sample men, refresher‐sample women, and current male partners. Across both phases of the study, men and women in the core sample were interviewed a maximum of nine times. Male partners contributed differing numbers of data points; once enrolled, they participated in all subsequent waves of TLT‐1, even if their index relationship ended (e.g., up to eight waves for male partners who enrolled at Wave 1; up to three for those who enrolled at Wave 6). During TLT‐2, women again recruited current male partners using tokens. Male partners from TLT‐1 were only included in TLT‐2 if they were still in a relationship with a female respondent. Male partners who appear in both the TLT‐1 and TLT‐2 datasets can be followed longitudinally across study phases using a single, unique identifier.

In 2015, TLT‐2 successfully re‐interviewed 80 percent of women and 71 percent of men in the core sample. A bivariate analysis of sample retention can be found in Table 2. Significant predictors of retention include: older age, not being enrolled in school at baseline, having less education, having been born in Balaka, ethnicity, and (for women) having ever been married. Photographs were used to confirm the identity of respondents during the study period.

## CONSENT

Respondents gave consent at the time they were recruited for the study, before each interview, and before biomarker collection. Unmarried men and women aged 15–17 in the core sample enrolled in the study only after the study team obtained informed consent from a parent or guardian and assent from the minors themselves.

## STUDY INSTRUMENTS

Malawian interviewers, trained extensively by the TLT research team and certified by Malawi's Ministry of Health to conduct HIV testing and counseling (HTC), conducted the interviews in Chichewa. Rather than following the standard household‐survey approach, which is often impacted by bystanders and interruptions (Trinitapoli and Weinreb 2015), interviews took place in private rooms at the TLT research center, located near Balaka's main market. Interviewers and respondents were matched on gender. During each wave of TLT‐1 and 2 respondents were asked questions from a core questionnaire about fertility behaviors and intentions, contraceptive use, relationships, perceived risk of HIV, HIV risk behaviors, HIV testing and treatment experiences, socioeconomic situation, childbearing, and health. In subsequent interviews, the core questionnaire was supplemented with in‐depth modules focusing on a range of topics (Table 3).

**Table 3:** TLT survey content and other components of study by wave

	TLT‐1									TLT‐2
	2009–2011								2012	2015
Core questionnaire	Wave 1	Wave 2	Wave 3	Wave 4	Wave 5	Wave 6	Wave 7	Wave 8	RS^a^	TLT‐2
B: Background	X								X	X
H: Health and happiness	X	X	X	X	X	X	X	X	X	X
F: Fertility preferences	X	X	X	X	X	X	X	X	X	X
M: Marriage	X	X	X	X	X	X	X	X	X	X
S: Sexual and romantic partnerships	X	X	X	X	X	X	X	X	X	X
A: HIV/AIDS	X	X	X	X	X	X	X	X	X	X
O: Exposures	X	X	X	X	X	X	X	X	X	X
X: Expectations	X	X	X	X	X	X	X	X	X	X
E: Economics	X	X	X	X	X	X	X	X	X	X
R: Religion	X	X	X	X	X	X	X	X	X	X
W: Education	X	X	X	X	X	X	X	X	X	X
I: Interviewer questions	X	X	X	X	X	X	X	X	X	X
K: Best friend	X	X		X	X	X	X	X		X
G: Shocks		X	X	X	X	X	X	X		
Q: Health services		X	X	X	X	X	X	X		X
TR: Travel		X	X	X	X	X	X	X	X	X
CR: Child roster	X	X	X	X	X	X	X	X	X	X
HH: Household roster		X	X	X	X	X	X	X		

Gender‐specific modules	Wave 1	Wave 2	Wave 3	Wave 4	Wave 5	Wave 6	Wave 7	Wave 8	RS^a^	TLT‐2
P: Pregnancy history (women only)	X	X	X	X	X	X	X	X	X	X
Z: Fathering history (men only)	X									X
PP: Pregnancy Questionnaire (pregnant women only)	X	X	X	X	X	X	X	X		
PX: Post‐Partum Questionnaire (post‐partum women only)		X	X	X	X	X	X	X		

	TLT‐1									TLT‐2
	2009–2011								2012	2015
Occasional modules	Wave 1	Wave 2	Wave 3	Wave 4	Wave 5	Wave 6	Wave 7	Wave 8	RS^a^	TLT‐2
U: Agreement	X			X						X
C: Flexibility of fertility preferences	X									X
V: Vignettes about HIV and fertility	X									X
D: Divorce	X									
J: Siblings and caregiving	X									
L: Residency and migration	X								X	
TY: Two years in the future		X								
RP: Relationship power			X		X					X
RH: Reproductive health				X						
RB: Risk behavior				X						
XE: Expectations expanded				X						
TO: Treatment optimism					X	X	X	X		X
RS: Relationship scripts					X					
L: Literacy							X			
N: Parent information								X	X	
NG: Digit Span Cognition Test										X
LM: London Measure of Unplanned Pregnancy										X
CS: Interactive ART Allocation Card‐Sort Exercise										X
T: Technology										X
TU: Time use										X

Biomarker data	Wave 1	Wave 2	Wave 3	Wave 4	Wave 5	Wave 6	Wave 7	Wave 8	RS^a^	TLT‐2
PG: Pregnancy biomarker (women only)	X	X	X	X	X	X	X	X	X	X
HIV: HIV biomarker^b^	R	R	R	R	R	R	R	R	X	X

a RS refers to the refresher sample interviewed following Wave 8.

b R indicates HIV biomarker was collected based on random assignment. See text for details.

X = Present at wave.

## ROSTERS

Respondents completed child rosters at baseline, in which they listed all the children they had given birth to or fathered, and full household rosters beginning at Wave 2. Interviewers updated these rosters at each subsequent wave, recording changes in the household's composition, including births and deaths.

## BIOMARKER DATA

At each wave, female respondents were asked to take an hCG pregnancy test. The acceptance rate ranged between 84 and 94 percent across waves; the main reason for refusal was menstruation. Table 4 presents the results across all waves of pregnancy testing by self‐reported pregnancy and identifies discrepancies between self‐report and biomarker result. For example, 3.6 percent of women who reported not being pregnant had a positive pregnancy test, and 4.1 percent of those who reported being pregnant had a negative pregnancy test. Overall, however, self‐reports of pregnancy and hCG test results are highly correlated (*r* = 0.81 when limited to pregnant/nonpregnant responses).

**Table 4:** Pregnancy test results by self‐reported pregnancy status, all waves

	Self‐reported pregnancy			
Pregnancy test	Not pregnant (N=11,037)	Pregnant (N=1,238)	Do not know (N=49)	Missing (N=42)
Not pregnant	83.8	4.1	71.4	57.1
Pregnant	3.6	87.2	28.6	23.8
Indeterminate	0.1	0.1	0.0	0.0
Refused for menses	4.2	0.0	0.0	2.4
Refused for other reason	8.3	8.6	0.0	16.7
	100%	100%	100%	100%

During TLT‐1, HIV testing and counseling was offered using a randomized experimental design in which the frequency of HIV testing was manipulated to allow researchers to examine the impact of HTC on a variety of behavioral and socioeconomic outcomes. At their baseline interview in 2009, core‐sample respondents were randomized into three groups, with male partners subsequently assigned to the same group as their nominating partner: one‐third would receive HTC at every interview, one‐third would receive HTC at Waves 4 and 8; and the final one‐third would not get tested at TLT until Wave 8. All respondents were offered HTC at TLT‐2. At Wave 1, HTC uptake among respondents offered testing was 85 percent for women and 78 percent for men (core sample and partners). In 2015, 93 percent of women and 91 percent of men consented to HIV testing. HTC used rapid HIV tests and followed the protocol set by the Malawi Ministry of Health and used by local clinics.

**Figure 2:**
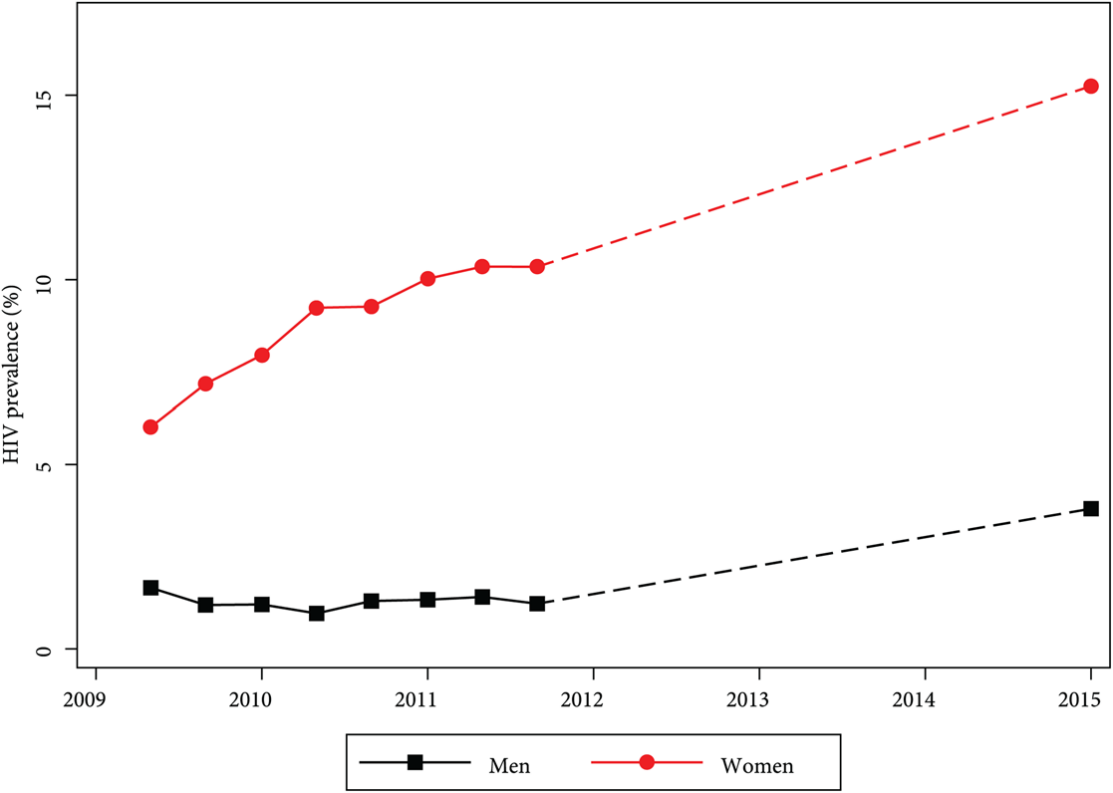
HIV prevalence among men and women in core sample, 2009–15

Among core‐sample respondents, HIV prevalence in 2009 (Wave 1) was 1.7 percent among men and 6.0 percent among women. By 2015 (TLT‐2), prevalence had increased to 3.8 percent for men and 15.2 percent for women (Figure 2). Although sizable, this increase in HIV prevalence is consistent with the known age and gender patterns of HIV prevalence that have characterized the country's epidemic for decades (NSO [Malawi] and ICF International 2016). Women's HIV incidence between the 2011 (Wave 8)/2012 (refresher) interviews and TLT‐2 (2015) when all respondents were offered testing was 1.58 per 100 person‐years (95% CI=1.21, 2.02).^1^

## ANCILLARY QUESTIONNAIRES

Following the core questionnaire and biomarker portions of the study, women with new and ongoing pregnancies completed a *pregnancy questionnaire* that asked about when and how they found out they were pregnant, how they reacted emotionally, prenatal health behaviors, and antenatal testing for HIV. In waves that followed a birth, women also completed a *postpartum questionnaire*, which asked about their delivery, postnatal behaviors, and infant's health. Over the course of the study, the TLT research team tracked respondents who had migrated, re‐interviewing those who had moved to other districts when possible. During TLT‐1, most tracking was done within the district; however, at Wave 8 and TLT‐2, migrants were followed throughout Malawi. When respondents truly could not be interviewed because of migration, interviewers conducted a *migration autopsy* with a family member or neighbor who knew the reasons for migration and the respondent's destination. Twenty‐one respondents in the core and refresher samples died during the study period; to gather information about these deaths, interviewers conducted *verbal autopsies* with a family member or neighbor.

## COUPLES AND PARTNERSHIP DATA

TLT data are designed to facilitate two types of dyadic data analysis. TLT collected data from women and men in relationships over time, capturing relationships as they formed, strengthened, and dissolved. Time‐varying, couple‐level linking files can be used to match data from TLT women to the corresponding data from the men they referred to the study. Additionally, all respondents were asked to report on up to three ongoing partnerships at each wave. These self‐reported partnerships can be followed longitudinally over TLT‐1 using a partnership‐link file—even if the partners themselves never enrolled in the study.

## VALUE OF THE DATA

TLT's distinguishing features differentiate it from other studies in the region and make it useful to public health researchers and social scientists working in a variety of disciplines. Specifically, the study includes:

- Detailed data from a population‐based sample in an HIV‐endemic context over time
- An intensive design with respondents interviewed every four months during TLT‐1
- A randomized, experimental approach to HIV testing and biomarkers for HIV status that mean researchers can study the impacts of knowledge of one's status as well as have data on respondents’ HIV status and incident HIV infections
- A biomarker for pregnancy
- Dynamic, dyadic data on couples over time

Key findings have centered on the changing HIV treatment context (Yeatman et al. 2013; Yeatman and Trinitapoli 2017), fertility preferences (Trinitapoli and Yeatman 2011; Yeatman, Sennott, and Culpepper 2013; Yeatman and Sennott 2014; Sennott and Yeatman 2018; Trinitapoli and Yeatman 2018), education and literacy (Smith‐Greenaway 2015; Frye 2017), young‐adult relationships (Frye and Trinitapoli 2015), and family dynamics (Bachan 2014; Trinitapoli, Yeatman, and Fledderjohann 2014). A publication list for TLT is available at https://scholar.google.com/citations?user=OFx9oPIAAAAJ&hl=en.

## LIMITATIONS

By design, TLT is a focused study of a particular period of the life course in a context of high HIV prevalence and early childbearing. As such, our study offers rich detail about men and women between the ages of 15 and 31 but cannot speak to behaviors and preferences outside this age range. Additionally, while the TLT sample is population‐based and representative of the catchment area in 2009, it is not nationally representative and the geographic connection weakens over time as some respondents migrate outside the catchment area. Data users without much knowledge of African societies may not fully perceive the very particular and simultaneously very typical features of Balaka. We urge interested data users to read classic and contemporary ethnographic accounts of daily life in this part of the world (Mitchell 1956; Peters 1997; Verheijen 2011). Additionally, the study is relatively small, and while there are up to nine waves of data from each respondent, the sample size may constrain certain analyses of subpopulations including men in the core sample.

Although TLT data are distinct in key ways, some users may want to compare TLT data to other high‐quality studies of adolescents and young adults in Malawi, such as the Marriage Transitions in Malawi project (MTM) (Beegle and Poulin 2017) and Malawi Schooling and Adolescent Study (MSAS) or to the Malawi Longitudinal Study of Families and Household (MLSFH) and the Malawi Demographic and Health Survey (MDHS), which cover a much wider age range.

## DATA AVAILABILITY

TLT data are available through Data Sharing for Demographic Research (DSDR) within ICPSR at the University of Michigan (https://www.icpsr.umich.edu/icpsrweb/DSDR/series/767).

Because of deductive disclosure concerns, all files are released under restricted‐use access agreements. More detailed information about the TLT study including survey instruments, documentation guides, a data key, a user forum, and links to open‐access versions of many publications are available on the TLT website: http://tsogololathanzi.uchicago.edu.

## Footnotes

1 Incidence was estimated for the 1,071 women with valid tests at both points in time. Exposure time was calculated as the difference in interview dates, converted to years.
